# Surgery

**Published:** 1905-11-04

**Authors:** 


### SURGERY.

**The Results of Prostatectomy.**—The results which an average surgeon will obtain from perineal or suprapubic prostatectomy, performed for senile enlargement of the prostate, will not be such as to justify that enthusiasm for the operation which appears to dominate the minds of some of those experts who are to the fore as writers on the subject. L. S. Pilcher [1] and G. S. Whiteside [2] have recently published papers which help one to discover what the real results of these operations have been. Pilcher points out that good results are reported to have been secured by the most diverse methods of operating. Yet the selection of a method of operating cannot be an unimportant matter in view of the anatomical relations and pathological changes of the gland. In England the suprapubic method of operating is preferred, but in America and France the perineal operations have been for the most part selected. R. Harrison [3] thinks that some degree of incontinence of urine is not a very uncommon event after the perineal operation. In the suprapubic operation the bladder and prostate are approached from their most accessible positions and there is little risk of hæmorrhage, of damaging the sphincter or retentive apparatus of the bladder, or of producing stricture of the urethra if free drainage is afforded. Many surgeons hold the view that the perineal route is the better if the prostate is of the small and hard type and if the thickness of the perineu m is only moderate. In considering the results of prostatectomies the following questions require answers: first, What is the mortality of the operations? secondly, What are the complications or untoward events which may follow the operation and mar the perfection of its results? thirdly, How frequently are these untoward events likely to attend prostatectomy? Discussing the mortality Pilcher writes: "At present it would seem that a surgeon must expect a death-rate after prostatectomy of from 5 to 10 per cent." He quotes, however, some recent statistics of interest as examples of unusual immunity from mortality, in some of which the mortality was below 5 per cent., and Freyer recently performed eighty-five operations by the suprapubic method with a mortality of only 5.5 per cent. There must of necessity be some mortality in these operations on prostatic patients from sepsis, renal insufficiency and the degenerations incidental to old age. The mortality is likely to be diminished by resorting to the operation in the earlier rather than in the later stages of the disease. Also, the more completely the obstruction is removed, the wider the door is left open, so to speak, for the exit of infective material, the less have subsequent septic complications to be feared. G. S. Whiteside discusses the mortality and results of perineal prostatectomy in three series of cases—namely, Group A, in which the cases, 37 in number, were operated upon by nine different surgeons; Group B, 38 cases reported by Murphy in 1904; and Group C, 150 cases reported by Young and Goodfellow (75 cases each). In Group A the mortality was about 20 per cent., in Group B 8 per cent., and in Group C 4 per cent. Perhaps the lower mortality in the latter two groups of cases is due to the greater skill and experience of the operators and to a more careful selection of cases. We think the mortality in Group A, operated upon by nine different surgeons, best illustrates the mortality which the average surgeon is likely to meet with. The next most important question to consider is that of the expectation of cure. Casper, quoted by Whiteside, says: "We call such prostatics cured as have remained *for years* without subjective symptoms, and when no considerable amount of residual urine remains in the bladder after micturition." Many published statistics of results are vitiated by the facts that the results quoted have not been reported at a sufficiently long period after the operation, they have been reported before they were known to have stood the test of time. Pilcher, in the paper we are considering, writes: "Prepossession and enthusiasm often lend a rose colour to the reports of results, and a more close scrutiny of real conditions may often elicit information as to attendant infirmities which really modify the result." But with these reservations the writer says that in more than 60 per cent. of the cases that have been subjected to total prostatectomy the ability to empty the bladder spontaneously has been restored and maintained permanently, so that the use of a catheter has no longer been necessary. Also, in a large proportion of the remaining cases, there has been a marked improvement in the obstructive symptoms, the residual urine has been less, cystitis has diminished, and the catheter has been easier to pass, and has been required less often. Whiteside, in his collected cases, referred to above, 238 in number, found that absolutely good results had been gained in 30 per cent. of them. All were operated on by the perineal method, and perineal prostatectomy is considered to be the best operation in most cases. The sequelæ, he points out, are numerous and troublesome. They include rectal fistula, urinary sinus, abscess, stricture at the bladder neck, incontinence, frequency of micturition, residual urine, epididymitis, sexual impotence, cystitis, vesical calculus, and urinary tuberculosis. Further, mistakes in diagnosis have not been uncommon, even in the practice of the most skilful. "Carcinoma, sarcoma, and syphilis have all proved stumbling-blocks." Whiteside pertinently asks, "If you yourself suffered from frequency, tenesmus, or even had been forced to lead a catheter life, would you eagerly embrace an operation offering a 30 per cent. chance of cure, a 7 per cent., or more, chance of death, and, more important still, a 50 per cent. chance that you would be no better, but have only exchanged one urinary difficulty for another? You would consider the operation a *very serious one*, and the outcome *doubtful*." Such being the case it is wrong to persuade patients that the operation is a safe one, and that if they survive it is certain to effect a cure. Pilcher considers individually these sequelæ which are apt to mar the results of prostatectomy. First there is impotence. If that portion of the prostate through which the ejaculatory ducts pass is removed, then injury of those ducts, entailing subsequent cicatricial obliteration, is almost inevitable. On the other hand, in many cases the hypertrophy affects the lateral lobes, and these may be enucleated without injuring the prostatic ducts. Horwitz has suggested that the impotence following prostatectomy may be due to the disturbance of the nervous mechanism of the gland rather than to injury of the ejaculatory ducts. In discussing this possible consequence of prostatectomy with those patients who still retain sexual vigour, they should be told that there is a very considerable possibility that the operation will deprive them of potency. In such cases a technique likely to preserve the continuity of the ejaculatory ducts should be selected, unless more imperative indications necessitate disregard of the sexual functions. In some cases urinary incontinence persists after prostatectomy. There may be urinary leakage when the patient moves about, or when asleep, or whenever the bladder becomes distended or the patient is fatigued. There is not reliable data to hand of the frequency with which this trouble occurs. Epididymitis and orchitis is fairly frequent during convalescence after prostatectomy from infection at the prostatic site. Suppuration may follow. Supraprubic, perineal, and recto-urethral fistulæ occasionally follow prostatectomy. The recto-urethral fistulæ may be the consequence of a wound of the rectum or of sloughing from local infection or drainage-tube pressure. Urethral stricture is an occasional, but not a usual, result of prostatectomy, even when the prostatic urethra has been extensively torn, nor even when this portion of the urethra has been entirely removed. For the avoidance of stricture the traumatism must not encroach upon any other portion of the urethra than the prostatic.

[1] Annals of Surgery, April 1905. [2] American Journal of Urology, August 1905. [3] Lancet, May 13, 1905.

